# Pandemic-related determinants of depression in Czech older persons: Evidence from the HAPIEE cohort: Andrea Dalecká

**DOI:** 10.1093/eurpub/ckac131.485

**Published:** 2022-10-25

**Authors:** A Dalecká, H Pikhart, N Čapková, M Bobák

**Affiliations:** 1 RECETOX, Faculty of Science, Masaryk University, Brno, Czechia; 2 Institute of Epidemiology & Health Care, University College London, London, UK; 3 National Institute of Public Health, Prague, Czechia

## Abstract

**Background:**

A number of studies reported higher levels of mental health issues during the COVID-19 pandemic but only minority of studies focused to assess changes in mental health against measurements taken before the pandemics. We investigated a change in depressive symptoms using repeated measurements and the impact of the pre-existing and COVID-19-related stressors in an ageing cohort in the Czech Republic.

**Methods:**

We used data on 2853 subjects (mean age 73 years) from the Czech part of the prospective HAPIEE cohort that participated on postal questionnaire surveys before (2017) and during the pandemic (autumn 2020 to early 2021). Participants reported their depressive symptoms using validated CESD-10 report tool. The impact of pre-existing stressors (age, sex, education, living alone, self-rated health, employment status, depression before pandemic), as well as pandemic-related stressors on change in depressive symptoms were tested using multivariable linear regression, after adjustment for age and potential confounders.

**Results:**

Compared with pre-pandemic period, there was a significant increase in depression score during the COVID-19 pandemic. The mean CESD-10 score increased from 4.92 to 5.37 (p < 0.001). Significantly larger increases in depressive score reported older persons (β = 0.073; p < 0.001) and those with poor self-rated health (β = 0.170; p < 0.001) in the fully adjusted model. Moreover, those who experienced social deprivation (β = 0.057; p < 0.001), death or hospitalization of a close person (β = 0.064; p < 0.001), delays in healthcare (β = 0.048; p = 0.005) and those who suffered from COVID-19 (β = 0.045; p = 0.008) also reported worsened depressive symptoms.

**Conclusions:**

This longitudinal study confirms important increase in depressive symptoms during the COVID-19 pandemic and contributes to identify pandemic-related risk factors. Interventions and future public health policies should address vulnerable individuals and population groups.

**Key messages:**

• Mental health worsened during the COVID-19 pandemic compared to previous years.

• Social deprivation, delays in healthcare and experiencing COVID-19 infection affected mental health of older people.

